# A Phase 1 randomized study on the safety and pharmacokinetics of OCS-05, a neuroprotective disease modifying treatment for Acute Optic Neuritis and Multiple Sclerosis

**DOI:** 10.1038/s41598-023-32278-0

**Published:** 2023-03-29

**Authors:** Pablo Villoslada, Mar Masso, Stephane Paris, Simon Hutchings, Annelize Koch

**Affiliations:** 1 Institut d’Investigacions Biomediques August Pi Sunyer, Rosello 149, 08036 Barcelona, Spain; 2 Bionure Farma/Accure Therapeutics SL, Barcelona, Spain; 3 Simbec-Orion, Merthyr Tydfil, UK

**Keywords:** Multiple sclerosis, Drug development

## Abstract

OCS-05 (aka BN201) is a peptidomimetic that binds to serum glucocorticoid kinase-2 (SGK2), displaying neuroprotective activity. The objective of this randomized, double-blind 2-part study was to test safety and pharmacokinetics of OCS-05 administered by intravenous (i.v.) infusion in healthy volunteers. Subjects (n = 48) were assigned to receive placebo (n = 12) or OCS-05 (n = 36). , Doses tested were 0.05, 0.2, 0.4, 0.8, 1.6, 2.4 and 3.2 mg/kg in the single ascending dose (SAD) part. In the multiple ascending dose (MAD) part, 2.4 and 3.0 mg/kg doses were administered with 2 h i.v. infusion for 5 consecutive days. Safety assessments included adverse events, blood tests, ECG, Holter monitoring, brain MRI and EEG. No serious adverse events were reported in the OCS-05 group (there was one serious adverse event in the placebo group). Adverse events reported in the MAD part were not clinically significant, and no changes on the ECG, EEG or brain MRI were observed. Single-dose (0.05–3.2 mg/kg) exposure (C_max_ and AUC) increased in a dose-proportional manner. Steady state was reached by Day 4 and no accumulation was observed. Elimination half-life ranged from 3.35 to 8.23 h (SAD) and 8.63 to 12.2 h (MAD). Mean individual C_max_ concentrations in the MAD part were well below the safety thresholds. OCS-05 administered as 2-h i.v. infusions of multiple doses up to 3.0 mg/Kg daily for up to 5 consecutive days was safe and well tolerated. Based on this safety profile, OCS-05 is currently being tested in a phase 2 trial in patient with acute optic neuritis (NCT04762017, date registration 21/02/2021).

## Introduction

Development of neuroprotective and remyelinating drugs is a high priority for central nervous system (CNS) diseases, including multiple sclerosis (MS)^1^. Among several approaches, activation of the trophic factor pathway has been pursued as a strategy to promote neuronal and axonal survival prior to damage as well as for oligodendrocyte precursor differentiation and myelin restoration. Some of the most prominent trophic factor pathways tested for neuroprotective activities include the neurotrophins family, such as nerve growth factor (NGF) and brain derived nerve factor (BDNF), insulin growth factor-1 (IGF-1), glial derived nerve factor, ciliary neurotrophic factor, or fibroblast growth factor (FGF)^2–4^. IGF-1 pro-survival effects are mediated by the activation of several kinases including serum glucocorticoid kinase (SGK), which leads to the activation of the transcription factor Forkhead Box O (FOXO) 3^5,6^. FOXO3 phosphorylation promotes its translocation outside the nucleus, and this triggers the expression of anti-apoptotic genes, anti-oxidant enzymes, and neuronal survival and differentiation genes, with the outcome of promoting neuronal survival and differentiation^7–9^. Therefore, SGK-FOXO3 activation is proposed as a target for the development of neuroprotective therapies^1,10^.

OCS-05 (previously known as BN201) is a neuroprotective and remyelinating drug^11^. OCS-05 activates SGK2, thereby activating FOXO3 pathway, which is known to be related to the neuronal survival response. In vitro and nonclinical in vivo studies indicate that OCS-05 has both neuroprotective and myelinating activity. OCS-05 has demonstrated activity in functional in vitro assays, promoting neuronal differentiation in the PC12 (rat pheochromocytoma) cell line, survival after induction of oxidative stress in the SH SY5Y (human neuroblastoma) cell line, and myelination by co-cultivating neurons with purified oligodendrocyte progenitor cells from rat^11^.

Currently OCS-05 has demonstrated neuroprotective properties by reducing the damage in the optic nerve and retina in in vivo models of acute optic neuritis (AON) such as lysolecithin-induced optic nerve demyelination, and in the experimental autoimmune encephalomyelitis model of MS. Moreover, OCS-05 showed activity in models of neurodegeneration such as the rat glaucoma model (by preventing retinal ganglion cell loss) and the in vitro model of amyotrophic lateral sclerosis (by rescuing motor neuron cells from death after nutritional stress), suggesting that the beneficial effects are mediated by neuroprotection and not immunomodulation^11^. Preclinical studies have shown that OCS-05 IV has a broad biodistribution, half-life 8-12 h and is able to cross the blood brain barrier by active transportation. Toxicology studies have identified presence of adverse events at high doses, either CNS (seizures) or cardiovascular (PR or QT prolongations) in rats and dogs. Based on such finding, a no observed adverse effect level (NOAEL) of 13.3 µg/mL was defined.

OCS-05 is being developed as a neuroprotective treatment for decreasing CNS damage and disability in acute relapses of MS such as AON (i.e., as acute neuroprotection) as well as for the long-term treatment of MS to prevent worsening disability (chronic neuroprotection). The objective of this study was to assess the safety, tolerability, and pharmacokinetics of i.v. OCS-05 in healthy volunteers in order to enable clinical testing in patients with AON, MS, or other neurodegenerative diseases.

## Results

### Safety

A total of 56 subjects were enrolled in the study (Table 1 and supplementary information). In the SAD part, out of the 40 subjects (4 cohorts of 8 subjects and 8 replacements), 32 subjects completed the SAD part. Eight subjects did not complete this study part, 7 requested early discontinuation due to personal or work constrains and 1 was discontinued pre-dose at the request of the Investigator for a PR interval that was persistently < 120 ms, none of them having experienced adverse events. For the MAD part, 16 subjects (2 cohorts of 8 subjects) were included. Fifteen subjects completed the MAD part, and one subject requested early termination for personal reasons after receiving all 5 doses of treatment. There were no replacement subjects during the MAD part.

**Table 1:** Demographics of subjects.

	SAD (N = 40)	MAD (N = 16)
Age (years), mean (SD)	33.0 (8.80)	30.4 (9.35)
Height (m), mean (SD)	1.751 (0.0890)	1.739 (0.0644)
Weight (kg), mean (SD)	79.61 (10.906)	78.16 (10.556)
BMI (kg/m^2^), mean (SD)	25.98 (3.282)	25.78 (2.652)
Gender, n (%) subjects		
Male	29 (72.5)	14 (87.5)
Female	11 (27.5)	2 (12.5)
Race, n (%) subjects		
White	40 (100.0)	15 (93.8)
Other	0 (0.0)	1 (6.3)

In the SAD part, there were a total of 10 TEAEs reported by 9 subjects (22.5%). The majority of subjects reported mild events, considered unrelated to OCS-05 (Table 2). There were no severe or serious TEAEs reported. Two (5.0%) subjects reported mild events that were considered of possible relationship to the treatment; 1 following placebo (mild pain [right elbow ache]); 1 following 1.6 mg OCS-05 (mild throat irritation). The events lasted approximately 3 days and 20 h respectively, no concomitant medication was administered and both subjects completely recovered. In addition, with the exception of the clinically significant Holter ECG results (a 10 beat run of aberrant beats with a rate of 140 bpm occurring at 02:01 h [classified as ventricular tachycardia], sinus rhythm with rates from 47 to 106 bpm) observed for subject 101 following placebo, there were no other clinically significant safety findings during the SAD part. Review of summary statistics revealed little difference between OCS-05 and placebo in the means and mean changes from baseline for 12-lead ECG parameters. No dose related effects of OCS-05 were observed during the SAD part and none of the subjects’ 12-lead ECG data met study stopping criteria.

**Table 2:** Overall summary of TEAEs – SAD part.

	Placebo (N = 14)	OCS-05								
		0.05 mg/kg (N = 6)	0.2 mg/kg (N = 6)	0.4 mg/kg (N = 6)	0.8 mg/kg (N = 6)	1.6 mg/kg (N = 6)	2.4 mg/kg (N = 6)	3.2 mg/kg (N = 6)	All (N = 32)	Overall (N = 40)
Number of TEAEs	2	3	1	0	1	1	1	1	8	10
Number (%) of subjects reporting at least one:										
TEAE	2 (14.3)	3 (50.0)	1 (16.7)	0 (0.0)	1 (16.7)	1 (16.7)	1 (16.7)	1 (16.7)	7 (21.9)	9 (22.5)
Serious TEAE	0 (0.0)	0 (0.0)	0 (0.0)	0 (0.0)	0 (0.0)	0 (0.0)	0 (0.0)	0 (0.0)	0 (0.0)	0 (0.0)
TEAE Leading to Withdrawal	0 (0.0)	0 (0.0)	0 (0.0)	0 (0.0)	0 (0.0)	0 (0.0)	0 (0.0)	0 (0.0)	0 (0.0)	0 (0.0)
Number (%) of subjects with TEAE by severity:										
Mild	2 (14.3)	2 (33.3)	1 (16.7)	0 (0.0)	0 (0.0)	1 (16.7)	0 (0.0)	1 (16.7)	4 (12.5)	6 (15.0)
Moderate	0 (0.0)	1 (16.7)	0 (0.0)	0 (0.0)	1 (16.7)	0 (0.0)	1 (16.7)	0 (0.0)	3 (9.4)	3 (7.5)
Severe	0 (0.0)	0 (0.0)	0 (0.0)	0 (0.0)	0 (0.0)	0 (0.0)	0 (0.0)	0 (0.0)	0 (0.0)	0 (0.0)
Number (%) of subjects with TEAE by relationship to IMP:										
Almost Definite	0 (0.0)	0 (0.0)	0 (0.0)	0 (0.0)	0 (0.0)	0 (0.0)	0 (0.0)	0 (0.0)	0 (0.0)	0 (0.0)
Probable	0 (0.0)	0 (0.0)	0 (0.0)	0 (0.0)	0 (0.0)	0 (0.0)	0 (0.0)	0 (0.0)	0 (0.0)	0 (0.0)
Possible	1 (7.1)	0 (0.0)	0 (0.0)	0 (0.0)	0 (0.0)	1 (16.7)	0 (0.0)	0 (0.0)	1 (3.1)	2 (5.0)
Unlikely	1 (7.1)	0 (0.0)	0 (0.0)	0 (0.0)	0 (0.0)	0 (0.0)	0 (0.0)	1 (16.7)	1 (3.1)	2 (5.0)
Unrelated	0 (0.0)	3 (50.0)	1 (16.7)	0 (0.0)	1 (16.7)	0 (0.0)	1 (16.7)	0 (0.0)	5 (15.6)	5 (12.5)

During the MAD part, a total of 9 TEAEs were reported by 5 (31.3%) subjects. The majority of subjects reported mild events, considered unrelated to treatment. No significant differences between TEAEs were observed between different OCS-05 doses. Review of the summary statistics revealed little difference between OCS-05 and placebo in the means and mean changes from baseline in 12-lead ECG parameters with similar values observed for placebo, 2.4 mg and 3.0 mg OCS-05. No subjects had 12-lead ECG data that met study stopping criteria. There was 1 serious adverse event (severe pneumonia; unlikely to be related to study medication) following placebo (Table 3). This subject had a cold that developed into a lower respiratory tract infection and subsequently pneumonia. The subject was hospitalised for approximately 4 days, during which the subject developed haematuria (moderate severity; unrelated to study medication) that resolved with sequalae from the pneumonia (fatigue and coughing) within approximately 9 days. At time of last contact, the pneumonia was reported to be improving.

**Table 3:** Overall summary of TEAEs–MAD part.

	Placebo (N = 4)	2.4 mg/kg OCS-05 (N = 6)	3.0 mg/kg OCS-05 (N = 6)	Overall (N = 16)
Number of TEAEs	7	1	1	9
Number (%) of subjects reporting at least one:				
TEAE	3 (75.0)	1 (16.7)	1 (16.7)	5 (31.3)
Serious TEAE	1 (25.0)	0 (0.0)	0 (0.0)	1 (6.3)
TEAE Leading to Withdrawal	0 (0.0)	0 (0.0)	0 (0.0)	0 (0.0)
Number (%) of subjects with TEAE by severity:				
Mild	2 (50.0)	0 (0.0)	1 (16.7)	3 (18.8)
Moderate	0 (0.0)	1 (16.7)	0 (0.0)	1 (6.3)
Severe	1 (25.0)	0 (0.0)	0 (0.0)	1 (6.3)
Number (%) of subjects with TEAE by relationship to IMP:				
Almost Definite	0 (0.0)	0 (0.0)	0 (0.0)	0 (0.0)
Probable	0 (0.0)	0 (0.0)	0 (0.0)	0 (0.0)
Possible	0 (0.0)	0 (0.0)	0 (0.0)	0 (0.0)
Unlikely	1 (25.0)	0 (0.0)	1 (16.7)	2 (12.5)
Unrelated	2 (50.0)	1 (16.7)	0 (0.0)	3 (18.8)

Overall, the most common adverse events were pain and upper respiratory infections. No other ECG abnormalities outside the one reported above were detected. Indeed, no abnormalities were observed in blood tests, EEG, or brain MRI.

### Pharmacokinetics

**Figure 1:**
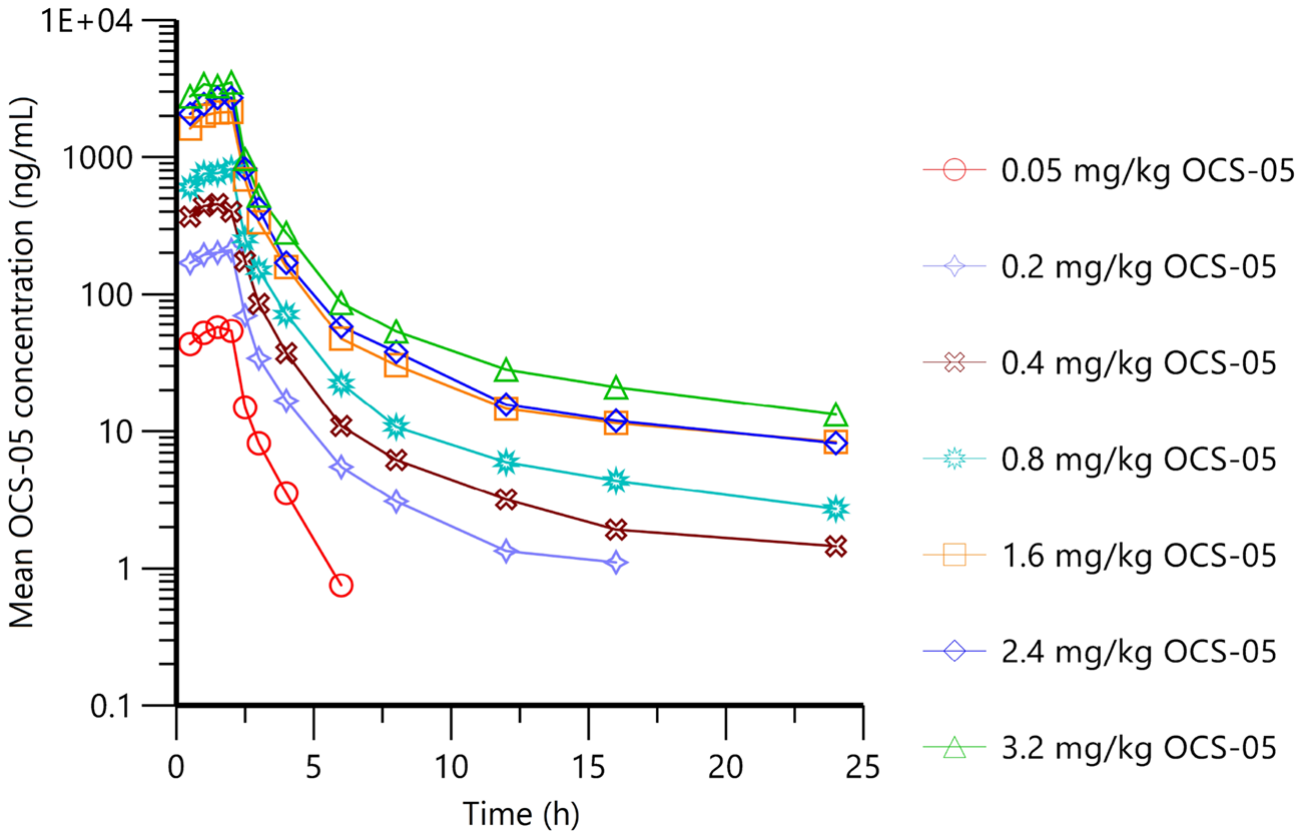
Summary of derived pharmacokinetic parameters following single dose administration of OCS-05 (i.v. infusion) to healthy male and female subjects.

In SAD part, C_max_ was reached at approximately 2 h post-dose (median T_max_ 1.5–2.0 h) (at or near the end of infusion) across the dose range, before declining in a biphasic manner over the remaining sampling time (to 24 h post-dose) (Fig. 1). C_max_ increased in a dose-proportional manner across the dose range (0.05–3.2 mg/kg) (Table 4), with no subject having a Cmax that exceeded the dose-escalation stopping criteria of 13.3 µg/mL (no observed adverse effect level [NOAEL] in the rat and dog 14-day study). Overall exposure (AUC_0-t_ and AUC_0-inf_) increased in a dose-proportional manner across the dose range (0.05–3.2 mg/kg), with AUC_%extrap_ (approximately 1–2%), indicating that the large majority of exposure was accounted for during the sampling period (to 24 h post dose). The T_1/2_ (geometric mean) ranged from 3.35 to 8.23 h across the dose range 0.2–3.2 mg/kg. The T_1/2_ (and associated parameters [k_el_, AUC_0-inf_, AUC_%extrap_, CL, Vz]) at the 0.05 mg/kg dose level was not calculable and the t_1/2_ at the 0.2 mg/kg dose level was shorter than that observed at the 0.4–3.2 mg/kg dose levels, at 3.35 h versus 6.84–8.23 h respectively. The “not-calculable” and shorter T_1/2_ (at the two lowest dose levels) is most likely the result of a poorly defined terminal elimination phase secondary to lower or below limit of quantitation plasma concentrations at the terminal end of the concentration time curve when compared to the higher dose levels. The CL (geometric mean) ranged from 23.6 to 28.8 L/h/kg. The Vz (geometric mean) ranged from 120.0 to 320.0 L/kg, suggesting that OCS-05 is widely distributed to perfused tissues.

**Table 4:** Summary of derived pharmacokinetic parameters following single dose administration of OCS-05 (i.v. infusion) to healthy subjects as means (SD).

	Geometric mean						
	OCS-05						
	0.05 mg/kg (N = 6)	0.2 mg/kg (N = 6)	0.4 mg/kg (N = 6)	0.8 mg/kg (N = 6)	1.6 mg/kg (N = 6)	2.4 mg/kg (N = 6)	3.2 mg/kg (N = 6)
C_max_ (ng/mL)	54.3 (20.7)	222 (48.5)	474 (38.5)	828 ((98.7)	2150 (231)	2750 (259)	3470 (833)
T_max_ (h)	1.50 (0.31)	2.00 (0.42)	1.55 (0.25)	2.00 (0.21)	1.50 (0.45)	1.75 (0.28)	1.75 (0.41)
AUC_0-t_ (h*ng/mL)	113 (39.4)	500 (133.8)	1110 (108.3)	1910 (222)	4960 (625)	6160 (567)	7930 (2319)
AUC_0-inf_ (h*ng/mL)	NC (NA)	528 (141.2)	1110 (75)	1910 (241)	5150 (745)	6550 (682)	8430 (2430)
AUC_%extrap_ (%)	NC (NA)	1.03 (0.54)	1.35 (0.54)	1.59 (0.21)	1.76 (0.39)	1.41 (0.74)	1.56 (0.33)
k_el_ (1/h)	NC (NA)	0.207 (0.12)	0.0893 (0.02)	0.0900 (0.01)	0.0865 (0.02)	0.0842 (0.04)	0.101 (0.01)
T½ (h)	NC (NA)	3.35 (2.96)	7.77 (2.72)	7.71 (1.13)	8.01 (1.43)	8.23 (4.59)	6.84 (0.58)
Vz (mL)	NC (NA)	120,000 (75,613)	298,000 (92,232)	320,000 (57,952)	298,000 (96,562)	280,000 (183,194)	259,000 (75,183)
CL (mL/h)	NC (NA)	24,900 (3989.7)	26,600 (3074)	28,800 (2519)	25,800 (4583)	23,600 (2020)	26,300 (6505)

**Figure 2:**
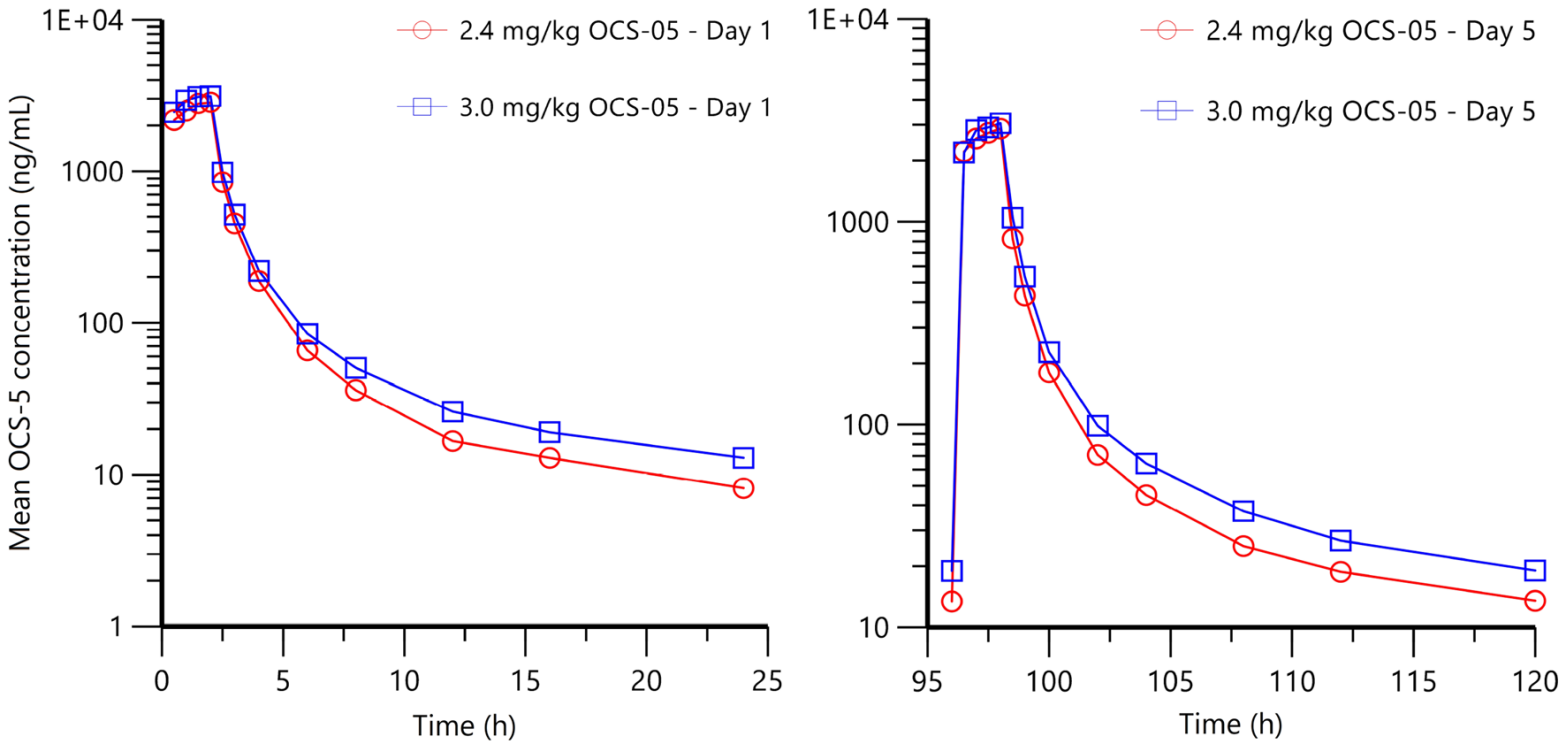
Summary of Derived Pharmacokinetic Parameters Following Multiple Dose Administration of OCS-05 (I.V. Infusion) to Healthy Male and female Subjects.

In the MAD part, C_max_ was reached at approximately 1.5–2 h post-dose (median T_max_) (at or near the end of infusion) on Day 1 and by 2 h post-dose on Day 5 following the 2.4 and 3.0 mg/kg doses, before declining in a biphasic manner over the remaining sampling time (to 24 h post-dose) (Fig. 2). No subject had a C_max_ that exceeded the dose-escalation stopping criteria of 13.3 µg/mL (NOAEL in the rat and dog 14-day study). The AUC_%extrap_ (approximately 3%), indicates that the large majority of exposure was accounted for during the sampling period (to 24 h post dose). Following the last dose (Day 5), OCS-05 was cleared from plasma with a t_1/2_ (geometric mean) of 8.63–12.2 h and CL_ss_ (geometric mean) of 27.7–27.9 L/h/kg. The V_ss_ (geometric mean) ranged from 60.2 to 63.6 L/kg, suggesting that OCS-05 is widely distributed to perfused tissues. Steady state was reached between Day 3–4 for 3.0 mg OCS-05 (Table 5). It is likely steady state was also reached by Day 3–4 for 2.4 mg OCS-05, however the geometric LSmean values on Day 4 for 2.4 mg OCS-05 were higher than expected in 1 subject, as a result of the Day 4 pre-dose PK sample being taken 2 min after the start of the infusion. This resulted in a pre-dose plasma concentration of 196 ng/mL being observed on Day 4 for this subject 037 compared to 5.6–19.2 ng/mL in the remaining subjects in the dosing group. The Day 4 pre-dose value was > 5% of the corresponding C_max_ (Day 5) for this subject, thus skewing the geometric LSmean ratio (90% CI) results.

**Table 5:** Summary of derived pharmacokinetic parameters following multiple dose administration of OCS-05 (i.v. infusion) to healthy subjects by Day 5 as means (SD).

	Geometric Mean	
	OCS-05	
	2.4 mg/kg (N = 6)	3.0 mg/kg (N = 6)
C_max_ (ng/mL)	2840 (587)	3080 (587)
T_max_ (h)	2.00 (0.258)	2.00 (0.258)
AUC_0-τ_ (h*ng/mL)	6450 (1560)	7170 (1560)
AUC_0-t_ (h*ng/mL)	6450 (1560)	7170 (1560)
AUC_0-inf_ (h*ng/mL)	6920 (1740)	7680 (1740)
AUC_%extrap_ (%)	3.27 (1.48)	2.92 (1.48)
k_el_ (1/h)	0.0567 (0.0212)	0.0804 (0.0212)
T½ (h)	12.2 (4.08)	8.63 (4.08)
Vss (mL)	60,200 (12,500)	63,600 (12,500)
CLss (mL/h)	27,900 (4720)	27,700 (4720)

In order to account for a subject who received an incorrect dose (2.85 mg/kg instead of 2.4 mg/kg), due to the administered dose being based on an incorrect weight of 77.1 kg (correct weight 65.0 kg), the PK analysis was also conducted excluding this subject using the supplementary PK set. The analysis of the supplementary PK set gave the same results as those described for the PK set above (data not shown).

## Discussion

The safety profile of OCS-05 in healthy subjects was favorable at the tested doses in the SAD and MAD parts of this study. With the exception of TEAEs that tended to be higher following placebo during the MAD, there were no other treatment or dose-related trends in safety parameters observed during either part of the study. Pharmacokinetic data for OCS-05 in the SAD and MAD parts of the study demonstrate that the C_max_ is reached within approximately 2 h of dosing, occurring at or near the end of the 2-h OCS-05 infusion. Following single-doses (0.05–3.2 mg/kg), overall exposure (C_max_ and AUC) increased in a dose-proportional manner. Following multiple dosing (2.4 and 3.0 mg) steady state was reached by Day 3–4 and no accumulation in exposure (C_max_ and AUC) was observed between first (Day 1) and last day (Day 5) of dosing. Finally, the half-life ranged from 3 to 8 h following single (0.2–3.2 mg/kg) and 12 and 8 h following multiple (2.4 and 3.0 mg/kg) dosing.

Preclinical pharmacokinetics studies of i.v. OCS-05 in rats and dogs have shown that a value of blood concentration (C_Blood_)/plasma concentration (C_Plasma_) between 0.8 and 1.2 was indicative of equal partitioning of OCS-05 into plasma and blood cells in any of the tested species. OCS-05 was found to have low binding to plasma proteins at the concentration evaluated (10 µM) and no species differences were observed in plasma protein binding^11^. Mean recovery values were between 85 and 89% in the plasma dialysis experiment performed relative to 6 h stability samples, which is indicative neither of significant binding to the dialysis equipment nor of solubility issues. Furthermore, OCS-05 was shown to be stable in rat, dog, and human plasma at 37ºC for 6 h. OCS-05 was not an inhibitor of tested cytochrome P450 (CYP) enzymes under the experimental conditions as the half maximal inhibitory concentration (IC50) values for OCS-05 against all 7 CYP isozymes were greater than the highest concentration assessed in this assay (i.e., 100 µM). This means that not one of the concentrations tested could inhibit the tested CYP isozymes. Results from this study indicate a linear relationship of pharmacokinetics parameters across species, including humans.

The doses in this study were chosen based on the human equivalent doses related to animal toxicology studies and based on the NOAEL. As such, we started with a dose × 10 under the NOAEL. The PK data indicate that Cmax is reached within approximately 2 h of single and 5-day multiple-dosing, occurring at or near the end of the 2 h OCS-05 infusion. The AUC%extrap (< 5%) indicates that the large majority of exposure was accounted for during the sampling period (to 24 h post-dose) following both single and multiple dosing. In the study, the t1/2 (geometric mean) ranged from 3.35 to 8.23 h across the 0.2–3.2 mg/kg OCS-05 dose range in Part A and 12.2 and 8.63 h for 2.4 and 3.0 mg/kg OCS-05 in Part B respectively. In both parts of the study, Vz (geometric mean) ranged from 120.0 (120,000 mL/kg) to 320.0 (320,000 mL/kg) L/kg in Part A and Vss (geometric mean) 60.2 (60,200 mL/kg) to 63.6 (63,600 mL/kg) L/kg in Part B, suggesting that OCS-05 is widely distributed to perfused tissues. This study has limitations such as not having collected cerebrospinal fluid samples for measuring OCS-05 concentrations after BBB crossing, not being able to reach the maximum Cmax planned because the AUC reached the stopping rule and not having validated biomarkers of target engagement and pharmacodynamics. Considering the PK profile from this study, the toxicology studies, and that OCS-05 is crossing the BBB by active transportation after IV infusion, we have determined that starting doses for the phase 2 trial being between 2–3 mg/kg (NCT04762017 and EUDRACT: 2020-003147-29). Regarding the mechanism of action of OCS-05 for promoting neuroprotection, we have previously shown that it activates SGK2^11^. SGKs are serine/threonine-protein kinases that target FOXO3, a ubiquitously expressed transcription factor that is highly expressed in CNS. SGKs are activated not only by post-translational modifications (phosphorylation, acetylation, methylation or ubiquitination) but also by hormones (mineralocorticoids such as aldosterone, and gonadotrophins such as FSH, IGF, CRH, HGF, and insulin)^12,13^, and by cytokines and trophic factors (TGFß, FGF, PDGF, IL-6, thrombin, endothelin)^14,15^. Vitamin D, PPARγ, glucose concentrations in serum, heat shock, changes in cell volume^16^, brain injury^17^, ultraviolet radiation and oxidative stress^18^ also activate SGKs.

Downstream effects of SGKs include the E3 ubiquitin ligase Nedd4-2, the FOXO3 and other kinases such as GSK-3B and NRDG1-2. In addition, SGKs regulates ion channels such as Epithelium ion channel (ENaC), CLC2, acid-sensing ion channel 1 (ASIC1), Kv1.3, Kv1.5, Kv4.3, ROMK1, TRVP5, CIC2, SCN5A, KCNE1/KCNQ1, KCNQ4, 4F2/LAT, GluR1 and GluR6, NHE3, as well as glucose transporters SGLT1, GLUT1, aminoacids transporter ASCT2, glutamate transporter EAAT1-5, CreaT, Na–K ATPase^18–20^. SGK2 also modulates ASIC1, an ion channel targeted by amiloride, which has been implicated in axonal degeneration in MS^21,22^. Also, SGKs inhibits the enzymes MEKK3, B-Raf, Nedd4-2, GSK3B. More, SGK modulates CREB, enhances the activity of NFkB, FOXO3a and ß-catenin.

SGK2 induces a broad spectrum of neurotrophic effects on neurons, including induction of neuronal hypertrophy, protection from neuron death and axonal degeneration and promoting axonal regeneration^23,24^. By upregulating glutamate transporters EAAT, SGK2 prevents excitotoxicity and provides neuroprotection^18^. Neuronal apoptosis is triggered by the GSK-3ß/ß-catenin pathway, a pathway that activates cell death and inhibits cell proliferation and that is suppressed by PI3K and SGK activation. Specifically, SGKs down-modulate GSK-3ß/ß-catenin by phosphorylating GSK-3ß on serine-9 and preventing cell death. After traumatic brain injury in mice, expression of SGKs is increased in the brain by Day 3 in neurons and in astrocytes, microglia, or oligodendrocytes^25^. Expression of SGKs is paralleled by expression of active caspase-3 and phosphor-GSK-3ß and ß-catenin. Therefore, activation of SGKs in response to brain damage down-regulates the GSK-3ß/ß-catenin pathway, preventing caspase-3 activation and neuronal death.

Phosphorylation by SGK induces FOXO3 translocation from the nucleus. FOXO3 contributes to the regulation of various processes such as cell cycle progression, cell size determination, cell death, cell differentiation, resistance to nutrient and oxidative stress, immune response, stress resistance, energetic metabolism and longevity^26^. Indeed, FOXO3 KO mice suffer autoimmunity due to defective Treg development^27^. Phosphorylation of FOXO3 in response to growth factors such as IGF-I, BDNF or NGF causes its exclusion from the nucleus, triggering the pro-survival and differentiation responses in neurons and glial cells^28–30^. Phosphorylation of FOXO3 in response to oxidative stress involves JNK and results in FOXO3 import in the nucleus^31^, and such effects on oxidative stress appears to prevail on the effect of growth factors^32^. The PI3K pathway protects neurons and axons for degeneration in a FOXO3-dependent manner and is related to superoxide dismutase (SOD) and catalase-increased expression^33^. Therefore, SGKs and FOXO3 have become a target for neuroprotective therapies for CNS diseases.

In summary, the results of this phase 1 study in healthy subjects with i.v. OCS-05 for up to 5 days showed a safe profile and linear pharmacokinetics, supporting testing it as a neuroprotective therapy for AON and MS. OCS-05 is currently being tested in a phase 2 trial in patients with AON of idiopathic origin or due to MS or MOG associated disease (MOGAD) (NCT04762017 and EUDRACT: 2020-003147-29).

## Methods

### Ethics statement

The study was conducted in accordance with the Helsinki Declaration of 1975, as revised in 2000, and approved by the Wales Research Ethics Committee and the UK Medicines and Healthcare products Regulatory Agency. The study was registered in EudraCT (number 2017-001202-14) on 06/11/2020. Researchers have disclosed to participants any potential conflict and the sponsor name. If the subject was willing to participate in the study, the informed consent form was signed and personally dated by the subject and the study staff member taking consent.

### Study design

This was a randomized, double blind, placebo controlled, 2-part single and multiple ascending dose study. The objectives were to assess the safety, tolerability, pharmacokinetics (PK), and pharmacodynamics of OCS-05 in healthy subjects. Inclusion criteria included healthy (as judged by the Investigator; no clinically significant abnormalities) male and female (non-pregnant, non-lactating) subjects between 18 and 55 years of age, with a body weight of ≥ 50.0 kg and ≤ 100 kg and body mass index (BMI) of 18 to 32 kg/m^2^, with no clinically significant history of previous allergy/sensitivity to OCS-05 or any of the excipients contained within the investigational medicinal product. Women of childbearing potential and male subjects with female partners of childbearing potential had to use one highly effective contraceptive precaution in addition to one acceptable contraceptive precaution (i.e., barrier precaution) from first dose until 3 months after last dose of study medication. See supplementary information for subject disposition, dosing, and assessments.

### Outcomes

The primary endpoint was the presence of Adverse Events (AEs), including and not limited to laboratory safety (biochemistry, hematology, and urinalysis); vital signs (supine systolic/diastolic blood pressure, pulse rate and body temperature); and 12-lead electrocardiogram (ECG: heart rate, PR interval, QRS duration, QT interval, QT interval corrected for heart rate using Bazett’s [QTcB interval] and Fridericia’s [QTcF interval] formulae). The secondary endpoints for this study were pharmacokinetics parameters: maximum concentration (C_max_); the time to maximum observed concentration (T_max_); elimination rate constant (k_el_); and terminal elimination half-life (t_1/2_). Other PK endpoints were the area under the concentration over time curve (AUC) from 0 to t, where t was the dosing interval (AUC_0- t_); AUC from the time of dosing to the time of the last measurable concentration (AUC_0-t_); AUC extrapolated to infinity (AUC_0-inf_); residual area (AUC_% extrapolated_); clearance (CL); clearance at theoretical steady state (CL_ss_); volume of distribution during terminal phase (V_z_); and the apparent volume of distribution at theoretical steady state (V_ss_).

### Treatments and doses

Subjects (n = 48) were randomly 1: 3 assigned to receive placebo (n = 12) or OCS-05 (n = 36). Due to the interlocking design where some subjects received more than one dose level, overall, 14 received placebo and 42 received OCS-05. In the single ascending dose part (SAD), doses administered with 2 h i.v. infusion were 0.05, 0.2, 0.4, 0.8, 1.6, 2.4 and 3.2 mg/kg. In the multiple ascending dose (MAD) part 2.4 and 3.0 mg/kg doses were administered with 2-h i.v. infusion for 5 consecutive days (Table 6). The starting dose of 0.05 mg/kg was decided as a very low dose based on toxicology studies and the higher dose of 3 mg/kg was defined based on the human equivalent dose with expected full efficacy while maintaining an adequate safety margin. Subjects returned for safety assessments at 24 h and 15 days after last dose.

**Table 6:** Summary of study design.

Study part	Screen	Cohort: Period (Dose)	Treatment period Duration	Follow-up
SAD	Day -28 to -2	SD1: Period 1 (0.05 mg/kg) Period 2 (1.6 mg/kg) SD2: Period 1 (0.2 mg/kg) Period 2 (3.2 mg/kg) SD3: Period 1 (0.4 mg/kg) Period 2 (2.4 mg/kg) SD4: Period 1 (0.8 mg/kg)	Day -1 to Day -3 Dose administered on Day 1 with a washout of at least 14 days between doses in Period 1 and 2	12 to 16 days after final dose
MAD	Day -28 to -2	MD1: Period 1 (2.4 mg/kg) MD2: Period 1 (3.0 mg/kg)	Day -1 to Day 7 Doses administered on Days 1—5	12 to 16 days after final dose

SD: Study Day.

### Statistical analysis

Safety population was defined as every subject randomized and treated at least once with either placebo or drug. All statistical analysis were performed using SAS^®^ version 9.3. The analysis sets included the safety set, the PK set, and the supplementary PK set. For each part of the study, demographic data were summarised descriptively (age, height, weight, and BMI) and by frequency (race and gender). Subject disposition and inclusion in analysis sets (safety, PK, supplementary PK) were also listed and summarised by frequency. AEs were coded using Medical Dictionary for Regulatory Activities version 21.0. The incidence of treatment emergent AEs (TEAE) was summarised by organ system, preferred term, severity, and relationship to study medication. Abnormal laboratory safety results were listed. Absolute values and change from baseline in laboratory safety parameters (including 24-h urinary sodium and potassium data), vital signs, and 12-lead ECG data were summarised descriptively. In addition, QTcB and QTcF values were summarised according to the following categories: i) for absolute values: QTcB/ QTcF ≤ 450 ms; 451 ≤ QTcB/ QTcF ≤ 480 ms; 481 ≤ QTcB/ QTcF ≤ 500 ms; QTcB/ QTcF > 500 ms; ii) for change from baseline: decreased/no change; QTcB/ QTcF increase ≤ 30 ms; 31 ≤ QTcB/ QTcF increase ≤ 60 ms; QTcB/ QTcF increase > 60 ms.

All OCS-05 concentrations and derived PK data were listed and individual and mean (arithmetic and geometric) concentration time data were plotted. Dose proportionality was assessed by performing a regression analysis of the log transformed C_max_, AUC_0-t_ and AUC_0-inf_ values versus the log transformed dose using the power model with a fixed effect for log(dose). For each parameter a point estimate and 95% confidence interval (CI) was calculated for the slope of the regression line. Geometric least squares mean (LSMeans) and associated 95% CI for derived PK parameters C_max_, AUC_0-t_ and AUC_0-inf_ were presented graphically by treatment.

For the Steady State analysis, for each dose level, log transformed trough concentration levels at pre dose each day (Day 2 through to Day 5) were subjected to a mixed-effects analysis of variance (ANOVA), with study day as a fixed effect and subject as a random effect, in order to establish whether and when steady state had been attained for each dose level. Back transformed ratios for the comparisons of each consecutive day (i.e., Day 3/Day 2) were presented along with corresponding 90% CI.

For the accumulation analysis, following logarithmic transformation, Cmax and AUC0-tau values were subjected to a mixed effects ANOVA for each dose level, including a fixed effect of study day and random effect of subject. The analysis included only subjects who had available data for both days within a treatment. Point estimates and 90% CI were constructed for the contrasts between Day 5 and Day 1 using the residual mean square error obtained from the ANOVA. The point and interval estimates were then back-transformed to give estimates of the ratios of the geometric LSMeans and corresponding 90% CI and the p-value for the test of the null hypothesis of non-equivalence. In addition, estimated geometric means were produced for each study day.

## Supplementary Information

Supplementary Information.

## Data Availability

The study protocol and statistical analysis plan are available in the supplementary material. The datasets generated and/or analysed during the current study are not publicly available due agreement restrictions but are available from the corresponding author on reasonable request.

## References

[1] Villoslada P, Steinman L (2020). New targets and therapeutics for neuroprotection, remyelination and repair in multiple sclerosis. Expert Opin. Investig. Drugs.

[2] Dremencov E, Jezova D, Barak S (2021). Trophic factors as potential therapies for treatment of major mental disorders. Neurosci. Lett..

[3] Savolainen M, Emerich D, Kordower JH (2018). Disease modification through trophic factor delivery. Methods Mol. Biol..

[4] Calkins DJ, Pekny M, Cooper ML, Benowitz L, Lasker IIoA, Glaucomatous Neurodegeneration P. (2017). The challenge of regenerative therapies for the optic nerve in glaucoma. Exp. Eye Res..

[5] Bhalla S, Mehan S, Khan A, Rehman MU (2022). Protective role of IGF-1 and GLP-1 signaling activation in neurological dysfunctions. Neurosci. Biobehav. Rev..

[6] Fernandez AM, Torres-Aleman I (2012). The many faces of insulin-like peptide signalling in the brain. Nat. Rev. Neurosci..

[7] Gui T, Burgering BMT. FOXOs: masters of the equilibrium. FEBS J 2021. 10.1111/febs.16221 PMC10078705 34610198

[8] Calissi G, Lam EW, Link W (2021). Therapeutic strategies targeting FOXO transcription factors. Nat. Rev. Drug. Discov..

[9] Stefanetti RJ, Voisin S, Russell A, Lamon S (2018). Recent advances in understanding the role of FOXO3. F1000Research.

[10] Brunet A, Park J, Tran H, Hu LS, Hemmings BA, Greenberg ME (2001). Protein kinase SGK mediates survival signals by phosphorylating the forkhead transcription factor FKHRL1 (FOXO3a). Mol. Cell Biol..

[11] Villoslada P, Vila G, Colafrancesco V (2019). Axonal and myelin neuroprotection by the peptoid BN201 in brain inflammation. Neurotherapeutics.

[12] Webster MK, Goya L, Firestone GL (1993). Immediate-early transcriptional regulation and rapid mRNA turnover of a putative serine/threonine protein kinase. J. Biol. Chem..

[13] Webster MK, Goya L, Ge Y, Maiyar AC, Firestone GL (1993). Characterization of SGK, a novel member of the serine/threonine protein kinase gene family which is transcriptionally induced by glucocorticoids and serum. Mol. Cell Biol..

[14] Waldegger S, Klingel K, Barth P (1999). h-sgk serine-threonine protein kinase gene as transcriptional target of transforming growth factor beta in human intestine. Gastroenterology.

[15] Cowling RT, Birnboim HC (2000). Expression of serum- and glucocorticoid-regulated kinase (sgk) mRNA is up-regulated by GM-CSF and other proinflammatory mediators in human granulocytes. J. Leukoc. Biol..

[16] Waldegger S, Barth P, Raber G, Lang F (1997). Cloning and characterization of a putative human serine/threonine protein kinase transcriptionally modified during anisotonic and isotonic alterations of cell volume. Proc. Natl. Acad. Sci. U.S.A..

[17] Imaizumi K, Tsuda M, Wanaka A, Tohyama M, Takagi T (1994). Differential expression of sgk mRNA, a member of the Ser/Thr protein kinase gene family, in rat brain after CNS injury. Brain Res. Mol. Brain Res..

[18] Lang F, Bohmer C, Palmada M, Seebohm G, Strutz-Seebohm N, Vallon V (2006). (Patho)physiological significance of the serum- and glucocorticoid-inducible kinase isoforms. Physiol. Rev..

[19] Baines D (2013). Kinases as targets for ENaC regulation. Curr. Mol. Pharmacol..

[20] Loffing J, Flores SY, Staub O (2006). Sgk kinases and their role in epithelial transport. Annu. Rev. Physiol..

[21] Arun T, Tomassini V, Sbardella E (2013). Targeting ASIC1 in primary progressive multiple sclerosis: Evidence of neuroprotection with amiloride. Brain.

[22] Vergo S, Craner MJ, Etzensperger R (2011). Acid-sensing ion channel 1 is involved in both axonal injury and demyelination in multiple sclerosis and its animal model. Brain.

[23] Chen X, Tagliaferro P, Kareva T, Yarygina O, Kholodilov N, Burke RE (2012). Neurotrophic effects of serum- and glucocorticoid-inducible kinase on adult murine mesencephalic dopamine neurons. J. Neurosci..

[24] David S, Stegenga SL, Hu P (2005). Expression of serum- and glucocorticoid-inducible kinase is regulated in an experience-dependent manner and can cause dendrite growth. J. Neurosci..

[25] Wu X, Mao H, Liu J (2013). Dynamic change of SGK expression and its role in neuron apoptosis after traumatic brain injury. Int. J. Clin. Exp. Pathol..

[26] Greer EL, Brunet A (2005). FOXO transcription factors at the interface between longevity and tumor suppression. Oncogene.

[27] Hagenbuchner J, Ausserlechner MJ (2013). Mitochondria and FOXO3: Breath or die. Front. Physiol..

[28] Brunet A, Bonni A, Zigmond MJ (1999). Akt promotes cell survival by phosphorylating and inhibiting a Forkhead transcription factor. Cell.

[29] Biggs WH, Meisenhelder J, Hunter T, Cavenee WK, Arden KC (1999). Protein kinase B/Akt-mediated phosphorylation promotes nuclear exclusion of the winged helix transcription factor FKHR1. Proc. Natl. Acad. Sci. U.S.A..

[30] Glauser DA, Schlegel W (2007). The emerging role of FOXO transcription factors in pancreatic beta cells. J. Endocrinol..

[31] Calnan DR, Brunet A (2008). The FoxO code. Oncogene.

[32] Wang Q, Li L, Xu E, Wong V, Rhodes C, Brubaker PL (2004). Glucagon-like peptide-1 regulates proliferation and apoptosis via activation of protein kinase B in pancreatic INS-1 beta cells. Diabetologia.

[33] Calixto A, Jara JS, Court FA (2012). Diapause formation and downregulation of insulin-like signaling via DAF-16/FOXO delays axonal degeneration and neuronal loss. PLoS Genet.

