# Supplementary material for: A Phase 1 randomized study on the safety and pharmacokinetics of OCS-05, a neuroprotective disease modifying treatment for Acute Optic Neuritis and Multiple Sclerosis
Source: Sci Rep. 2023 Mar 29;13:5099. doi: 10.1038/s41598-023-32278-0 (PMC10060579; doi:10.1038/s41598-023-32278-0)
Supplement: Supplementary file 1 — Supplementary Information. [file 41598_2023_32278_MOESM1_ESM.docx]

**Supplementary information**

## Study Design and Treatments

This will be a randomized, blinded, placebo-controlled study of OCS-05 in adults ages 18-55.

This is a Phase 1 randomized, double-blind, single and multiple ascending dose study consisting of a single ascending dose (SAD) part (Part A) and a multiple ascending dose (MAD) part (Part B) to assess the safety, tolerability, PK and PD of ascending single and multiple doses of OCS-05. Each treatment (OCS-05 or placebo) will be administered via a 2‑hour intravenous (IV) infusion.

A total of 48 healthy subjects will be enrolled in 4 single dose groups and in 2 multiple dose groups. Before each dose escalation, the sponsor and Principal Investigator must agree on a next dose level as described in a dose escalation report (DER), which will be sent to the MHRA for a statement of no objection. The dose levels may be lowered depending on the safety or available PK observations at each dose level.

Part A (SAD)

SAD Study will have an at least biweekly dose escalation setup, in which the first cohort will be dosed at a low dose of 0.05 mg/kg.

In this first-in-human study, the subjects participating at each dose level (8 volunteers) will be dosed according to a sentinel dosing design to ensure optimal safety. Initially, 2 subjects will be dosed on the same day: 1 with OCS-05 and 1 with placebo. If the safety and tolerability results of the first 24 hours following dosing for the initial subjects are acceptable to the PI, the other 6 subjects (5 active and 1 placebo) may be dosed over 3 consecutive days, meaning that in all groups of the SAD part a maximum of 2 subjects will be dosed per day.

After the first dose is completed and safety and PK-PD data are available, we would apply stopping criteria to proceed with subsequent doses. Further doses are intended to reach a C_max_ always below 13.3 µg/mL, which is significantly lower than the C_max_ associated with side-effects in rodents and dog studies.

The following treatments are planned to be administered:

Period 1: Group SD1 a single IV infusion of 0.05mg/kg OCS-05 (n=6) or placebo (n=2)

Group SD2 a single IV infusion of 0.2 mg/kg OCS-05 (n=6) or placebo (n=2)

Group SD3 a single IV infusion of 0.4 mg/kg OCS-05 (n=6) or placebo (n=2)

Group SD4 a single IV infusion of 0.8 mg/kg OCS-05 (n=6) or placebo (n=2)

Period 2: Group SD1 a single IV infusion of 1.6 mg/kg OCS-05 (n=6) or placebo (n=2)

Group SD2 a single IV infusion of 3.2 mg/kg OCS-05 (n=6) or placebo (n=2)

Group SD3 a single IV infusion of 6.0 mg/kg OCS-05 (n=6) or placebo (n=2)

Group SD4 a single IV infusion of TBC* mg/kg OCS-05 (n=6) or placebo (n=2)

**Optional; 7 Dose levels are planned but an optional 8th dose level may be evaluated if required*

The alternating panel design of Group SD1, SD2, SD3 and SD4 is presented schematically in Figure 1 below.

| **Group** | | | **Period 1** | | |  | **Period 2** | | |  | | |
| --- | --- | --- | --- | --- | --- | --- | --- | --- | --- | --- | --- | --- |
| **SD1:** | SD1 (0.05 mg/kg) | 🡪 | |  |  | | | SD1 (1.6 mg/kg) |  | |  |  |
| **SD2:** |  | SD2 (0.2 mg/kg) | | 🡪 |  | | |  | SD2 (3.2 mg/kg) | |  |  |
| **SD3:** |  |  | | SD3 (0.4 mg/kg) | 🡪 | | |  |  | | SD3 (6.0 mg/kg) |  |
| **SD4:** |  |  | |  | SD4 (0.8 mg/kg) | | | 🡪 |  | |  | SD4 (TBC mg/kg) |

Figure 1 Flowchart for Single Ascending Dose Administration of OCS-05 in Part A

*Study Schedule*

*Volunteers will stay from Day -1 to Day 2 at the clinical center for pretreatment procedure on Day -1, treatment on Day 1 and 24h after treatment observation until Day 2.*

*A follow-up will be done on Day 8. See table 1 for schedule of assessments.*

Part B (MAD)

Part B will be conducted in 2 multiple dose (MD) groups (Group MD1 and MD2) of 8 healthy subjects each. In each group, subjects will be randomized to receive OCS-05 (6 per group) or placebo (2 per group) once daily for 5 consecutive days (from Day 1 to Day 5). The dose levels to be administered will be based on the safety, tolerability and PK results of Part A. Group MD1 can only be started if a higher dose level in the SAD was well tolerated. Before dose escalation to Group MD2, the safety and tolerability of the subjects in Group MD1 will be assessed up to 24 hours post-last-dose.

The following treatments are planned to be administered:

Group MD1: once daily IV infusion of xx mg/kg* OCS-05 (n=6) or placebo (n=2) for 5 consecutive days

Group MD2: once daily IV infusion of xx mg/kg* OCS-05 (n=6) or placebo (n=2) for 5 consecutive days

Table 1 Schedule of Assessments – Part A (SAD Part)

|  | **Assessment Period^1^** | | | | | | |
| --- | --- | --- | --- | --- | --- | --- | --- |
| **Visit** | **Screening** | **Pre‑Treatment** | | **Treatment** | | | **Follow‑up** |
| **Study Day** | **‑28 to ‑2** | **Day ‑1** | **Day 1 (Pre‑dose)** | **Day 1** | **Day 2** | **Day 3** | **Day 12‑16 days** |
| Confinement^1^ |  | x | x | x | x | x |  |
| Ambulatory | X |  |  |  |  |  | X |
| Admission |  | X |  |  |  |  |  |
| Informed Consent | x |  |  |  |  |  |  |
| Discharge |  |  |  |  |  | X |  |
| Physical examination^2^ | x |  |  |  |  | x^2^ | X |
| Medical History | x | X |  |  |  |  |  |
| Demographics | x |  |  |  |  |  |  |
| Body weight and height (including BMI calculation)^3^ | x | X^3^ |  |  |  |  | X^3^ |
| β‑hCG pregnancy test (females only) | x | X |  |  |  |  | X |
| FSH (females only) | x |  |  |  |  |  |  |
| Serology (HIV, HBsAg, HCV) | x |  |  |  |  |  |  |
| Drug and alcohol screen | x | X |  |  |  |  |  |
| Clinical laboratory^4^ | x | X | X | X | X | X | X |
| 12‑lead ECG^5^ | x | x | x | X | X | X | X |
| Telemetry^6^ |  |  | X | X |  |  |  |
| Holter monitoring^7^ |  |  | X | X |  |  |  |
| EEG monitoring^8^ | x |  | X | X |  |  |  |
| Vital signs and body temperature^9^ | x | X | x | X | X | X | X |
| Eligibility check | x | X | X |  |  |  |  |
| Randomisation |  |  | X^10^ |  |  |  |  |
| Study drug administration^11^ |  |  |  | x |  |  |  |
| Infusion site reaction^12^ |  |  | X | X | x | X | X |
| Blood sampling for PK^13^ |  |  | X | x | x |  |  |
| Blood sampling for PD^14^ |  |  | X | X |  |  |  |
| Blood sample for genotyping^15^ |  |  |  | X |  |  |  |
| Previous and concomitant medication | x | x | x | x | x |  | X |
| Adverse event monitoring |  | x | x | x | x |  | X |
| Brain MRI scan^16^ | X |  |  |  |  |  | X |
| Urine collection for sodium/potassium monitoring^17^ |  | X | X | X |  |  |  |

β‑hCG: β‑human chorionic gonadotropin; BMI: body mass index; ECG: electrocardiogram; EEG: electroencephalogram; FSH: follicle stimulating hormone; HBsAg: hepatitis B surface antigen; HCV: hepatitis C virus; HIV: human immunodeficiency virus; PD: pharmacodynamic(s); PK: pharmacokinetic(s); SAD: single ascending dose

1 Subjects will be in the clinic for 2 periods, each period from Day ‑1 until completion of the morning assessments on Day 3. There will be a washout period of at least 14 days between drug administrations. Subjects will return for follow‑up on Day 12‑16 of Period 2.

2 At discharge only a symptom directed physical examination.

3 For each subject, the intravenous dose of BN201 or placebo will be based on bodyweight as measured on Day ‑1.

4 Clinical laboratory (haematology, serum chemistry and urinalysis): at screening, Day ‑1, at pre‑dose and 1 h post‑start‑infusion on Day 1, at 24 and 48 h post‑start‑infusion and at follow‑up. Troponin I will be measured in the serum chemistry samples at pre‑dose and at 24 h post‑start‑infusion.

5 12‑lead ECG: single at screening and at follow‑up. Triplicates on Day 1: at ‑0.25, ‑0.5 and ‑0.75 h pre‑dose, at 0.25, 0.5, 0.75, 1 h, 1.25, 1.5, 1.75, 2 h (immediately after the end of infusion), 2.5, 3, 4, 5, 6, 8, 12, 24, 48 h post‑start‑infusion. During the treatment period, ECGs are to be taken during the last 5 mins of a 10‑min period of rest in the supine position, just before blood sampling and vital signs.

6 Telemetry: recorded on Day 1 from at least 1 h before start infusion until at least 8 h post‑start‑infusion.

7 Holter monitoring: recorded on Day 1 from at least 1 h before start infusion until at least 24 h post‑start‑infusion.

8 EEGs will be obtained at screening and on Day 1 from 0.5 h before start infusion until at least 4 h post‑start‑infusion.

9 Vital signs (supine systolic and diastolic blood pressure, pulse): at screening, Day ‑1, at pre‑dose and 0.5, 1, 2, 4, 12, 24 h post‑start‑infusion on Day 1, and at follow‑up. Body temperature: at screening, Day ‑1, at pre‑dose on Day 1, 24 h post‑start‑infusion, and at follow‑up.

10 Only in Period 1.

11 Intravenous infusion with BN201 or placebo for 120 mins. Note: start of infusion is t=0 for all assessments.

12 Infusion site reaction: at pre‑dose on Day 1, and at 1, 2, 8, 24, 48 h post‑start‑infusion, and at follow‑up.

13 Blood sampling for PK of BN201 in plasma: at pre‑dose and 0.5 h, 1, 1.5, 2 h (immediately after the end of infusion), 2.5, 3, 4, 6, 8, 12, 16, 24 h post‑start‑infusion on Day 1.

14 Blood sampling for PD of BN201: at pre‑dose on Day 1 and at 0.5, 1 and 2 h (immediately after the end of infusion) post‑start infusion.

15 A blood sample for genotyping will be taken post-dose on Day 1 of period 1 only

16 A brain MRI scan will be performed during the screening period which will also serve as a baseline measurement.

17 Urine will be collected for sodium/potassium monitoring from admission to the clinic on Day -1 until pre-dose, then from dosing until 24 h post dose.

Table 2 Schedule of Assessments – Part B (MAD Part)

|  | **Assessment Period^1^** | | | | | | | | | | |
| --- | --- | --- | --- | --- | --- | --- | --- | --- | --- | --- | --- |
| **Visit** | **Screening** | **Pre‑Treatment** | | **Treatment** | | | | | | | **Follow‑up^1^** |
| **Study Day** |  | **Day ‑1** | **Day 1 (Pre‑ dose)** | **Day  1** | **Day  2** | **Day  3** | **Day  4** | **Day  5** | **Day  6** | **Day 7** | **Day 15 (±2 days)** |
| Confinement^1^ |  | x | x | x | X | X | X | X | X |  |  |
| Ambulatory | X |  |  |  |  |  |  |  |  |  | X |
| Admission |  | X |  |  |  |  |  |  |  |  |  |
| Informed Consent? | X? |  |  |  |  |  |  |  |  |  |  |
| Discharge |  |  |  |  |  |  |  |  | X | X |  |
| Physical examination^2^ | X |  |  |  |  |  |  |  |  | X^2^ | X |
| Medical History | X | X |  |  |  |  |  |  |  |  |  |
| Demographics | x |  |  |  |  |  |  |  |  |  |  |
| Body weight and height (including BMI calculation)^3^ | X | X^3^ |  |  |  |  |  |  |  |  | X^3^ |
| β‑hCG pregnancy test (females only) | X | X |  |  |  |  |  |  |  |  | X |
| FSH (females only) | X |  |  |  |  |  |  |  |  |  |  |
| Serology (HIV, HBsAg, HCV) | X |  |  |  |  |  |  |  |  |  |  |
| Drug and alcohol screen | X | X |  |  |  |  |  |  |  |  |  |
| Clinical laboratory^4^ | X | X | X |  |  | x |  | x | x | x | X |
| 12‑lead ECG^5^ | X | x | X | X | X | X | X | X | X | X | X |
| Telemetry^6^ |  |  |  | X |  |  |  | X |  |  |  |
| Vital signs, and body temperature^7^ | X | X | X | X | X | X | X | X | X | X | X |
| Eligibility check | X | X | X |  |  |  |  |  |  |  |  |
| Randomisation |  |  | X |  |  |  |  |  |  |  |  |
| Study drug administration^8^ |  |  |  | x | X | X | X | X |  |  |  |
| Infusion site reaction^9^ |  |  | X | X | X | X | X | X | X |  | X |
| C‑SSRS questionnaire | X |  |  | x |  |  |  |  | x |  | x |
| QST and VAS^10^ |  | X |  |  |  |  |  | X |  |  |  |
| Blood sampling for PK^11^ |  |  | X | X | X | X | X | X | X |  |  |
| Blood sampling for PD^12^ |  |  | X | X | X | X | X | X | X |  |  |
| Blood sample for genotyping^13^ |  |  |  | X |  |  |  |  |  |  |  |
| Previous and concomitant medication | X | x | x | x | X | X | X | X | X |  | X |
| Adverse event monitoring |  | x | x | x | X | X | X | X | X |  | X |
| EEG^14^ | X |  |  | X |  |  |  | X |  |  |  |
| Holter monitoring^15^ |  |  | X | X |  |  |  | X |  |  |  |
| MRI Scan^16^ | X |  |  |  |  |  |  |  |  |  | X |
| Urine collection for sodium/potassium monitoring^17^ |  | X | X | X | X | X | X | X | X |  |  |

β‑hCG: β‑human chorionic gonadotropin; BMI: body mass index; C‑SSRS: Columbia‑Suicide Severity Rating Scale; ECG: electrocardiogram; FSH: follicle stimulating hormone; HBsAg: hepatitis B surface antigen; HCV: hepatitis C virus; HIV: human immunodeficiency virus; MAD: multiple ascending dose; PD: pharmacodynamic(s); PK: pharmacokinetic(s); QST: quantitative sensory testing; VAS: visual analogue scale

1. Subjects will be in the clinical research centre from the morning of Day ‑1 until completion of the morning assessments on Day 7.
2. At discharge only a symptom directed physical examination.
3. For each subject, the intravenous dose of BN201 or placebo will be based on bodyweight as measured on Day ‑1.
4. Clinical laboratory (haematology, serum chemistry and urinalysis) at screening, on Day ‑1, at pre‑dose on Days 1, 3 and 5, on Day 6 , Day 7 , and at follow‑up. Troponin I will be measured in the serum chemistry samples at pre‑dose on Day 1 and on Day 6.
5. Standard single 12‑lead ECG: at screening, from Day 1 to Day 4 at pre‑dose and 2 h post‑start‑infusion, on Day 5 at pre‑dose and at 0.5 h, 1 h, 1.5, 2 (immediately after the end of infusion), 3, 4, 6, 8, 16, 24 h post‑start‑infusion, and at follow‑up. During treatment period the ECGs are to be taken after 10 mins rest in the supine position, before blood sampling and vital signs.
6. Telemetry: On Day 1 and Day 5 from at least 1 h pre‑dose until at least 12 h post‑start‑infusion.
7. Vital signs (supine systolic and diastolic blood pressure, pulse): at screening, on Day ‑1, at pre‑dose and 0.5, 1, 2, 4, 12, 24 h post‑start‑infusion on Days 1 and 5, at pre‑dose on Days 2, 3 and 4, and at follow‑up. Body temperature at screening, on Day ‑1, at pre‑dose on Days 1, 3 and 5, on Day 6, and at follow‑up.
8. Intravenous infusion with BN201 or placebo for 120 mins once daily for consecutive 5 days (from Day 1 to Day 5). Note: start of infusion is t=0 for all assessments.
9. Infusion site reaction: at pre‑dose from Day 1 to Day 6, and at follow‑up.
10. QST for mechano‑sensitivity and VAS for spontaneous pain: at baseline on Day ‑1 and on Day 5 (close to t_max_, to be decided based on logistical feasibility)
11. Blood sampling for PK of BN201 in plasma: at pre‑dose and 0.5 h, 1 h, 1.5, 2 (immediately after the end of infusion), 2.5, 3, 4, 6, 8, 12, 16, 24 h post‑start‑infusion on Days 1 and 5, and at pre‑dose on Days 3 and 4.
12. Blood sampling for PD of BN201: on each dosing day (Day 1 to Day 5) at pre‑dose and at 0.5, 1 and 2 h (immediately after the end of infusion) post‑start infusion and on Day 6.
13. A blood sample for genotyping will be taken post-dose on Day 1 of period 1 only.
14. EEGs will be obtained at screening and on Day 1 and Day 5 from 0.5 h before start infusion until at least 4 h post‑start‑infusion. EEG monitoring only performed for Part B Cohort 2 if indicated from results from Part B Cohort 1.
15. Holter monitoring: recorded on Day 1 and Day 5 from at least 1 h before start infusion until at least 24 h post‑start‑infusion.
16. A brain MRI scan will be performed during the screening period which will also serve as a baseline measurement
17. Urine will be collected for sodium/potassium monitoring from admission to the clinic on Day -1 until pre-dose, then for a 24 h period following each dose.
